# The Impact of Postpartum Depression on the Early Mother-Infant Relationship during the COVID-19 Pandemic: Perception versus Reality

**DOI:** 10.3390/ijerph21020164

**Published:** 2024-01-31

**Authors:** Misty C. Richards, Camila A. Ferrario, Ying Yan, Nicole M. McDonald

**Affiliations:** Jane and Terry Semel Institute for Neuroscience and Human Behavior at UCLA, Department of Psychiatry and Biobehavioral Sciences, David Geffen School of Medicine, University of California, Los Angeles, CA,90095, USA; cferrario@mednet.ucla.edu (C.A.F.); yying4466@gmail.com (Y.Y.); nmcdonald@mednet.ucla.edu (N.M.M.)

**Keywords:** postpartum depression, perinatal depression, mother-infant bonding, mother–infant play interactions, oxytocin

## Abstract

Postpartum depression (PPD) can interfere with the establishment of affective bonds between infant and mother, which is important for the cognitive, social–emotional, and physical development of the child. Rates of PPD have increased during the COVID-19 pandemic, likely due to the added stress and limited support available to new parents. The present study examined whether parenting-related stress, perceived bonding impairments, the quality of observed mother–infant interactions, and salivary oxytocin levels differ between depressed and non-depressed mothers, along with differential impacts of COVID-19 on depressed mothers. Participants included 70 mothers (45 depressed, 25 controls) with infants aged 2–6 months. All data were collected remotely to ease participant burden during the pandemic. Depression was associated with experiences of heightened parenting-related stress and bonding difficulties. These differences were not observed during mother–infant interactions or in salivary oxytocin levels. Differences in COVID-19-related experiences were minimal, though depressed mothers rated slightly higher stress associated with returning to work and financial impacts of the pandemic. Findings highlight the importance of early intervention for PPD to mitigate long-term effects on mothers, children, and families. Additionally, they underscore the need for early intervention to support the developing mother–infant dyad relationship during this crucial time.

## 1. Introduction

Perinatal depression is a common public health problem that, if untreated, may have significant downstream consequences for child development and psychopathology. Approximately 20% of mothers suffer from depression during the perinatal period, defined as the time period during pregnancy and up until one year after delivery [1]. Adequate treatment of perinatal depression is of paramount importance, as it can have significant negative consequences for both mother and child. Infants raised by depressed mothers show early disruptions in social and emotional development, including diminished security of attachment to their mothers and ability to self-regulate, as well as greater vulnerability to psychopathology including depression and anxiety disorders [2,3]. The transmission of risk from depressed mothers to their children involves genetic influences as well as detached parenting, the latter negatively affecting the attachment relationship [3]. Postpartum depression (PPD), specifically, can hinder the establishment of affective bonds between infant and mother, interfering with maternal perception and interpretation of their child’s communication signals [4]. In addition, new mothers may struggle with their identity as caregivers, including the associated guilt surrounding their perceived inadequacies, which may be particularly enhanced through the lens of depression. 

Rates of PPD increased during the COVID-19 pandemic as the safety surrounding social connection was compromised [5,6]. When new mothers returned home from shortened hospitalizations, they faced increased isolation and less social support, two potent and well-established risk factors for PPD [7]. Furthermore, several services provided during maternal hospitalizations were pruned away, including education in infant care and breastfeeding, staff-supported labor recovery, and careful planning for ongoing management of mental health needs [7]. New mothers faced with structural inequities such as limited access to perinatal mental health treatment, unpaid and inadequate maternity leave, and unaffordable childcare during the pandemic highlighted the fractures in an already broken system [6]. As such, it is critical that we improve our understanding of the early emergence, context, and treatment of PPD. 

While the impact of PPD on the mother–child relationship and child development is relatively well established [8,9,10,11], biological factors, such as oxytocin, that may underlie or contribute to these challenges are less clear. Oxytocin is a nine-amino-acid neuropeptide synthesized in the hypothalamus that plays an important role in birth and lactation while also serving as a neurohormonal substrate for mammalian social bonding [12]. Oxytocin promotes positive parenting behaviors such as social synchrony, which refers to the mother’s capacity to observe and adapt to micro-level shifts in their infant’s socio-affective signals and enter a matched social dialogue with their infant [13,14]. Oxytocin’s role in maternal depression is less clear. A systematic review focused on oxytocin and depression in the perinatal period found some evidence that higher oxytocin levels were associated with lower depressive symptomatology [15]. Endogenous maternal oxytocin has also been shown to functionally buffer the effects of depression on offspring, potentially serving as a biological moderator of the parent–child relationship in the context of maternal depression [16]. On the other hand, Guintivano et al. [17] compared women with PPD with a community sample with no diagnosis, finding no significant difference in plasma oxytocin levels between the groups after controlling for adverse life events and breastfeeding status. More targeted research needs to be carried out, though it is clear that investigating endogenous neuroendocrine substrates like oxytocin has the potential to inform treatment while elucidating the underlying biological mechanisms of social connectedness. 

The current study sought to better understand the early emergence of PPD in the context of the COVID-19 pandemic in a group of depressed mothers of young infants who were not yet receiving medication treatment. We collected subjective measures of the level of depression symptoms, parenting-related stress, and perceived bonding. Objective measures related to bonding and the early parent–child relationship were also obtained, including observations of mother–infant free play and salivary oxytocin levels. We asked the following research questions: (1) Do self-reported (parenting-related stress, perceived bonding impairments), observed (mother-infant play behavior), and biological (salivary oxytocin level) measures of parenting function and mother–infant bonding differ between depressed and non-depressed mothers of 2- to 6-month-old infants? (2) Does the level of depression symptoms correlate with measures of parenting function and bonding? (3) Do depressed mothers report more COVID-19-related worries and impacts than non-depressed mothers? We hypothesized that depressed mothers would report higher levels of parenting stress, perceive bonding as more impaired, and have lower observed dyadic interaction quality and parent sensitivity. Given the mixed literature on oxytocin levels and depression, these analyses were more exploratory. 

## 2. Materials and Methods

### 2.1. Participants and Procedures

Participants included 70 mothers (depressed: *n* = 45, controls: *n* = 25) with healthy infants aged 2–6 months, who were followed through to 12 months of age. Here, we report data from the baseline study visit. Mothers were recruited between the years 2020 and 2023. Depressed mothers were informed about the study by their obstetrician or psychiatrist at UCLA. If they agreed to be contacted, a research team member reached out with further information and screening questions. Recruitment efforts for the control group also involved posting flyers in online platforms, such as parent groups, and relied on word-of-mouth referrals from participating mothers. This study included depressed mothers who screened positive on the Edinburgh Postnatal Depression Scale (EPDS) with a score of 10 or higher [18] and had received a confirmatory diagnosis of major depressive disorder with peripartum onset from a reproductive psychiatrist. Depressed mothers were not included if they were taking antipsychotic or mood-stabilizing medications or had a past diagnosis of psychotic or bipolar spectrum disorder. Depressed mothers were also excluded if they had been on selective serotonin reuptake inhibitor (SSRI) medication for more than two weeks prior to the baseline visit. Mothers in the control group scored a 9 or lower on the EPDS and reported no history of any psychiatric diagnoses. All infants were born full term and did not have any major medical issues. Socio-demographic factors of the sample are presented in Table 1. Participants in the depressed and control groups were comparable in education, race, and ethnicity, but control participants were more likely to be married and depressed participants were more likely to formula-feed their infant. Infant age and gender composition were similar across groups. 

All study procedures were approved by the UCLA Institutional Review Board. Informed consent was obtained from all participants prior to study participation. The study was conducted in accordance with the Declaration of Helsinki, and the protocol was approved by the Ethics Committee of IRB #18-001456. Study participation occurred remotely via Zoom. During the first one-hour virtual visit, a highly trained research associate recorded a 10 minute mother–infant play session, administered several questionnaires, completed a medical history interview, and oversaw the self-administration of saliva collection for oxytocin analysis. 

### 2.2. Measures

#### 2.2.1. Edinburgh Postnatal Depression Scale (EPDS)

The EPDS [18] is a widely used screener in perinatal care that comprises 10 questions rated on a Likert scale from 0 to 3. EPDS scores of 10 and above suggest heightened depressive symptoms. This measure was used as a screener, as well as a measure of the level of depression symptoms.

#### 2.2.2. Postpartum Bonding Questionnaire (PBQ) 

The PBQ [19] was used to measure the perceived quality of mother–infant bonding. It includes 25 self-report items with four subscales. The subscales include Impaired Bonding, Rejection and Pathological Anger, Infant-Focused Anxiety, and Incipient Abuse. A higher score on each subscale indicates a greater level of impairment [20]. The PBQ has good test–retest reliability. All subscales were analyzed, with the exception of Incipient Abuse, which was uncommon in this sample. 

#### 2.2.3. Parenting Stress Index, Fourth Edition, Short Form (PSI-4-SF)

The PSI-4-SF [21] is a widely utilized 36-item self-report questionnaire designed to measure the degree of stress within the parent–child dyad. This study focused on three subscales to measure distinct aspects of parenting stress. The Parental Distress Scale gauges the extent to which parents feel competent, restricted, conflicted, supported, and/or depressed in their parental role. The Parent–Child Dysfunctional Interaction Scale evaluates parental satisfaction with their child and the quality of interactions. The Difficult Child Scale measures how parents perceive the ease or difficulty of caring for their child. The Total Stress Scale provides an overall indication of the stress level experienced by individuals in their role as parents, combining the parent and child domains. The PSI-4-SF has high internal consistency and good test–retest reliability. All subscales were included in the analyses.

#### 2.2.4. COVID-19 Questionnaire 

To assess participants’ subjective experiences related to the impact of COVID-19, participants were asked to rate their agreement with four statements using a Likert scale ranging from 1 (strongly disagree) to 5 (strongly agree). This questionnaire was developed independently for descriptive purposes, and its reliability and validity were not established.

#### 2.2.5. Mother–Infant Play Interaction

Mother–infant free play sessions occurred in the home environment and were recorded via Zoom for a total of 10 min. Mothers were instructed to “Play with your baby as you normally do”. A trained examiner observed with her camera off to ensure adequate administration and videorecording of the task. Free play data were available for all but one participant (*n* = 69).

*Free play coding*. Interactions were coded offline using the Coding Interactive Behavior system [22]. This coding system was developed by Dr. Ruth Feldman and shows good test–retest reliability, construct validity, external validity, and predictive validity across normative and high-risk populations [23]. For the purposes of this study, 17 items focused on parenting behavior (Parent Supportive Presence removed), 9 items on infant behavior (added a Child Response code), and 5 dyadic behavior items were coded (removed Child-Led Interaction and Parent-Led Interaction codes). Each item was rated on a scale from 1 to 5, with half-point increments allowed. To consolidate the results for analyses, we averaged the ratings across several items based upon prior work [23]. Composite scores showed high levels of internal consistency, including Maternal Sensitivity (acknowledging, parent gaze, vocal appropriateness/clarity, positive affect, appropriate range of affect, affectionate touch, resourceful, consistency of style; α = 0.92), Child Social Engagement (alert, child initiation, child vocalization/verbal output, child positive affect, child gaze, child response; α = 0.85), and Dyadic Quality (dyadic reciprocity, adaptation regulation, fluency; α = 0.92).

*Reliability*. Two research associates were trained for reliability by the senior author (McDonald), who was trained in the system by the developer. Coding was completed by a research assistant blind to group status and other participant information, with 29% of interactions double-coded by a second research associate. Intraclass correlations (ICCs; single measures, absolute agreement) revealed high reliability across composite scores used in the analyses: Maternal Sensitivity (0.93), Child Social Engagement (0.93), and Dyadic Quality (0.93).

#### 2.2.6. Salivary Oxytocin

Saliva samples were obtained from the mother, and the collection was timed to occur at least 30 min after breastfeeding and 30 min before the next anticipated feeding session for nursing mothers [24]. Saliva collection kits were mailed to each participant’s home prior to the first virtual visit. The saliva sample was collected following the 10 min free play session. Samples were initially stored in participants’ freezers until a research associate picked them up. Samples were transferred on ice to the lab, where they were stored at −80 °C before being transported on dry ice to the Salimetrics SalivaLab. Samples were assayed in triplicate at the Salimetrics SalivaLab (Carlsbad, CA, USA) using an electrochemiluminescence (ECL) method developed and validated for saliva by Salimetrics. The average coefficient of variation for all samples tested was <20–30% and exceeds the applicable NIH guidelines for Enhancing Reproducibility through Rigor and Transparency. The sample test volume was 25 μL of saliva per determination. The assay has a lower limit of sensitivity of 8 pg/mL and a dynamic range from 8 pg/mL to 1000 pg/mL. 

A total of 70 salivary oxytocin samples were collected remotely during the Zoom visit, with 67 sent for analysis and 63 yielding oxytocin data. Three samples were lost prior to analysis due to a lack of participant response while arranging for pick up (*n* = 1) or being misplaced by the participant (*n* = 2). Four samples were sent for analysis but unable to be analyzed due to an insufficient quantity of saliva (*n* = 1) or a lack of detectable oxytocin (*n* = 3).

### 2.3. Data Analysis Plan

Preliminary analyses included visual and statistical examinations of variables of interest to inform the selection of appropriate statistical tests. Correlations between self-report scales (PSI and PBQ) and free play composite scores were then assessed, as were differences in oxytocin levels related to maternal breastfeeding status. For Aim 1, we examined group differences (depressed vs. controls) using *t*-tests for most variables (exceptions based on preliminary analyses noted below). For Aim 2, we assessed associations of variables of interest with the level of depression symptoms using Pearson’s correlations (see exception below). Results are presented with and without correction for multiple comparisons via the False Discovery Rate (FDR) method [25]. Exploratory analyses were conducted to follow up on unexpected findings.

## 3. Results

### 3.1. Preliminary Analyses

The Maternal Sensitivity rating was negatively skewed (most mothers were rated high), with a non-normal distribution, so nonparametric tests were used for this variable. All other variables had acceptable kurtosis and skewness values. 

Subscales of the self-report measures were moderately to highly correlated (*r*s = 0.44−0.77). Free play composite scores were also correlated. Maternal Sensitivity ratings were positively associated with Child Social Engagement, *r*(68) = 0.51, *p* < 0.001, and Dyadic Quality, *r*(68) = 0.68, *p* < 0.001. Child and dyad ratings were also highly correlated: *r*(69) = 0.78, *p* < 0.001.

Salivary oxytocin levels varied by feeding status in the expected direction, *F*(2, 60) = 5.86, *p* = 0.005. Mothers who exclusively breastfed had the highest oxytocin levels (M = 23.63, SD = 16.32), followed by those who fed both formula and breastmilk (M = 14.50, SD = 11.08), with the lowest levels in mothers who formula-fed (M = 7.17, SD = 9.33). Given these findings and group differences in feeding status between the groups, feeding status was controlled for in the oxytocin-related analyses.

### 3.2. Group Differences in Depressed vs. Control Mothers

**Figure 1:**
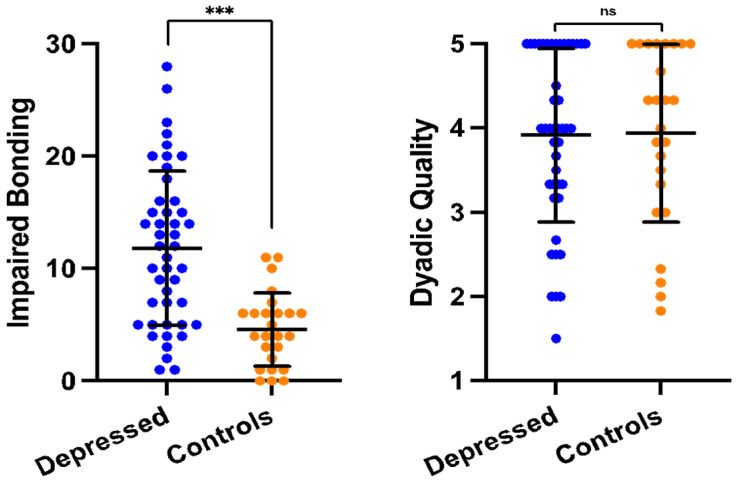
Perceived bonding versus observed dyadic interaction quality. *Note*. Depressed mothers reported higher levels of impaired bonding than non-depressed mothers. However, differences in the quality of dyadic interactions were not observed in a 10 min mother–child free play situation. *** *p* < 0.001, ns = not significant.

Findings from the group difference analyses are detailed in Table 2. Self-report measures of parenting-related stress (PSI) and perceived bonding (PBQ) differed significantly between groups (PSI *d*s = 7.14–9.48; PBQ *d*s = 2.85–5.87). Unexpectedly, the observed mother, child, and dyadic behaviors during free play did not differ (see Figure 1). Likewise, salivary oxytocin levels were equivalent between groups after controlling for feeding status (similar results were found without controlling for feeding, *p* = 0.744). All significant findings survived FDR correction.

Given the lack of findings for the composite scores, we explored whether there were group differences in individual aspects of observed parent, infant, and dyadic behavior. For the parent variables, mothers in the depressed group were rated as slightly higher in Parent Depressed Mood, *p* = 0.028 (uncorrected); no other variables significantly differed. None of the infant variables significantly differed, although children of depressed mothers tended to be rated *higher* on positively oriented variables (e.g., Child Positive Affect: depressed M = 4.49, SD = 0.98; controls M = 3.88, SD = 1.39; *p* = 0.06) and *lower* on negatively-oriented variables (e.g., Fatigue: depressed M = 1.47, SD = 0.97; controls M = 2.10, SD = 1.40; *p* = 0.05) than infants of control mothers. Dyadic ratings did not differ between groups. 

### 3.3. Correlations with Level of Depression Symptoms

The level of depression symptoms (EPDS) was highly positively correlated with parenting-related stress (PSI Total Stress), *r*(69) = 0.73, *p* < 0.001. Analysis of PSI-4-SF subscales indicated a particularly high correlation with parental distress, *r*(69) = 0.86, *p* < 0.001, and moderate correlations with reports of the quality of parent–child interactions, *r*(69) = 0.51, *p* < 0.001, and perceptions of the difficulty of parenting their baby, *r*(69) = 0.45, *p* < 0.001. Moderate positive correlations were also found between the level of depression symptoms and reports of impaired bonding, *r*(69) = 0.56, *p* < 0.001; feelings of rejection and anger with their baby, *r*(69) = 0.50, *p* < 0.001; and anxiety about their baby, *r*(69) = 0.50, *p* < 0.001 (PBQ). All findings survived FDR correction.

As above, observed mother–infant interaction variables did not correlate with the level of depression symptoms: Maternal Sensitivity, *r*(68) = −0.03, *p* = 0.76; Child Social Engagement, *r*(68) = 0.17, *p* = 0.17; and Dyadic Quality, *r*(68) = 0.03, *p* = 0.80. Salivary oxytocin levels were also not correlated with depression levels, *r*(62) = 0.20, *p* = 0.12 (controlled for feeding status; similar results when uncontrolled, *r* = 0.11).

We also explored the degree to which maternal perceptions of parent–child interactions corresponded with observed dyadic quality. Overall, the observed quality of play interactions did not correlate with perceived parent–child interaction quality (PSI), *r*(68) = −0.17, *p* = 0.17, or perceived bonding (PBQ), *r*(68) = −0.04, *p* = 0.78. When exploring within-group correlations, depressed mothers continued to show no correlation between perceived and observed interaction quality, *r*(43) = −0.13, *p* = 0.40, while the correlation for mothers in the control group approached significance in the expected direction *r*(24) = −0.38, *p* = 0.06 (higher reported dysfunctional interaction associated with lower dyadic interaction quality).

### 3.4. COVID-19 Experiences

Given the timing of our study, we also asked mothers about their COVID-19-related experiences (see Table 3). No significant group differences were found, though depressed mothers tended to report higher levels of stress related to returning to work and financial impacts of the pandemic, suggesting we may have lacked power to detect significant differences.

## 4. Discussion

This study examined the role of postpartum depression in mothers’ perceptions of the parent–child relationship, and the degree to which depression symptoms seemed to impact parenting behavior during play between mothers and their infants. Given the role of oxytocin in mother–infant bonding, we also investigated whether salivary oxytocin levels varied by depression status. Finally, we investigated whether depressed mothers reported heightened stress related to the COVID-19 pandemic than mothers who were not experiencing depression.

### 4.1. Perceived versus Observed Mother–Infant Interactions

As expected, depressed mothers reported higher levels of parenting-related stress and impairments in bonding compared to controls. We also detected a continuous relationship in which higher levels of depression symptoms corresponded with more parenting-related stress, particularly parental distress, impaired bonding, feelings of rejection and anger with their baby, and anxiety about their baby. In contrast, there were no differences in maternal sensitivity or dyadic interaction quality during observations of mother–infant interactions. These results support the idea that postpartum depression can fuel negative cognitive distortions, reinforcing fears of inadequacy, overwhelm, and frustration in new mothers. When micro-failures are reiterated in the process of matrescence (i.e., “I can’t calm my baby”, “I can’t feed my baby”), the mother does not perceive the reinforcement of feeling competent and confident as she parents [26]. Ultimately, depressed mothers may not always demonstrate the overt functional impairments they subjectively feel in this new role, though they may perceive each challenge as further evidence of inability. Over time, these repeated experiences leave a new mother vulnerable to the risk of negative mirroring, namely that of a bad child paired with a bad mother [27]. 

The lack of observable differences in mother–infant interactions was unexpected given the well-documented impacts of maternal depression on the parent–child relationship [8,9,10,11]. There are several potential explanations for this pattern of findings. The mothers in our study had relatively young infants (2–6 months, mean age = 3 months) and much of the prior work finding differences in interactive behavior was conducted slightly later in life [28]. We saw glimpses of trend-level differences in infant behavior that may be emerging, with the infants of depressed participants showing slightly more positive social behaviors, such as positive affect. While this finding should be interpreted with caution, it may be that infants of depressed mothers learn to first try harder to get their mother’s attention, before eventually giving up and becoming more passive [29]. 

Contextually, conclusions were drawn about the quality of dyadic interactions based on observations of a relatively brief and low-stress play interaction. Overall ratings of maternal sensitivity skewed high (see Figure 1). It may be that mothers in both groups felt compelled to perform, knowing that they were being closely observed and evaluated. Results may reflect a depressed mother’s ability to reconstitute for short periods of time and may not serve as a representative sample of dyadic interaction quality throughout the day. It is also possible that the global coding system used did not capture micro-level differences in interactive behavior between the groups [30]. The research associate leading the virtual visits informally noted qualitative differences in maternal behavior during interactions with her versus interactions with their baby, suggesting that these mothers were able to “turn on” temporarily for their babies.

Despite these caveats, this gap between perception and observed reality suggests a promising opening for early intervention. The depressed mothers in our study often showed the capacity to respond sensitively and positively to their child, even if this did not align with their perceptions. For instance, the observed quality of the interaction correlated more highly with reported interaction difficulties for non-depressed mothers than the depressed mothers. Behavioral interventions that guide mothers with PPD to see what they are doing right, while also helping them to more consistently pick up on their child’s social cues, have the potential to limit the impact of PPD on the developing relationship, as well as child outcomes [31]. Intervening directly to lessen maternal depression as early as possible, such as with the use of SSRIs, is also necessary to decrease maternal suffering and increase the mother’s capacity to engage in dyadic work.

### 4.2. Oxytocin Findings

While salivary oxytocin levels varied in the expected direction based on breastfeeding status (mothers who exclusively breastfed had the highest oxytocin levels), oxytocin levels did not differ based on depression diagnosis or symptoms regardless of whether we controlled for feeding status. In the literature, the majority of published plasma oxytocin studies support an inverse relationship between endogenous oxytocin levels and depressive symptoms, though results are inconsistent and causality has not been determined. A systemic review found that, of the twelve studies focused on endogenous oxytocin, eight suggested an inverse relationship between plasma oxytocin levels and depressive symptoms, three found no significant relationship, and one actually showed a positive relationship [32]. Taken together, discrepancies in oxytocin levels could be attributed to differences in laboratory measurement procedures, including extraction protocols, consideration of covariates, and individual study limitations. Given publishing biases toward positive findings, it is also possible that the number of studies that have failed to find a difference in oxytocin levels in maternal depression is underestimated. 

Our study utilized salivary oxytocin rather than plasma oxytocin, largely due to its non-invasive, convenient collection protocol during COVID-19. Understandably, our sample of new mothers preferred in-home collection compared to presenting to an outside laboratory, limiting unnecessary exposures. While salivary oxytocin levels are more than 10-fold lower than plasma values [33], our samples were analyzed in triplicate using an ECL method, which is highly sensitive, specific, and rapid, with a low background signal compared to the commonly used enzyme-linked immunosorbent assay technique [34,35,36]. Furthermore, given that reference ranges for normal endogenous oxytocin levels in postpartum women have not been established, the most useful comparisons come from relative oxytocin differences within the same sample rather than absolute values [32]. More refined research needs to be carried out on this prosocial neuropeptide, controlling for variables such as exposure to exogenous oxytocin (i.e., Pitocin, which is often used to strengthen uterine contractions during labor); specific collection, storage, and extraction protocols; and the timing of collection to make meaningful conclusions about its contribution to social connectedness in the PPD population. 

### 4.3. COVID-19 Experiences

Somewhat surprisingly, we did not find clear differences in COVID-19-related experiences between mothers with depression and without, though the depressed group reported slightly higher levels of stress related to returning to work and the financial impacts of the pandemic. The United States is the only high-income country in the world without federal protections for paid leave for working women who give birth. Currently, only 15% of workers in the United States have access to paid maternity leave, with rates skewed toward women of higher socioeconomic status [37]. As paid leave is associated with a lower likelihood of postpartum depression and maternal stress—which supports the findings of this study—mandating paid leave to working mothers could reduce disparities in maternal and child health [5]. New mothers faced with structural inequities enhanced by the pandemic such as limited access to care, unpaid and unprotected maternity leave, and unaffordable childcare are forced to operate in a system that does not serve them. Supporting policy changes that address these inequities can mitigate maternal depressive symptoms and improve mental health and well-being for mothers, children, and families for generations to come.

### 4.4. Limitations and Future Directions

We acknowledge that our findings are preliminary and in need of replication in a larger sample. Recruiting postpartum women during a pandemic proved to be challenging, though we found that participants were pleased with the fact that they did not have to leave their home to contribute to the study. While our sample was relatively small, our lack of findings with regard to the play interactions and oxytocin levels did not appear to be due to a lack of power, given the clear similarity of the data across groups. A lack of power may have affected our ability to detect subtler differences in COVID-19 experiences as we observed trend-level effects here. 

Given that data were collected remotely, including the observation of dyadic interactions between mother and baby, it is possible that more subtle cues and gestures were missed and not incorporated into the coding. On the other hand, a home interaction may have been more comfortable and truer-to-life than a lab-based interaction. We used a low-stress play interaction prompt. Future studies would benefit from including additional scenarios to detect potential differences in behavior that may arise during stress activation. We also only included mothers, given the specific focus on postpartum depression. The inclusion of other important caregivers, such as fathers, would have provided important context for understanding the broader social environment. 

Oxytocin is an inherently unstable molecule, requiring freezing temperatures below 2 °C to prevent degradation. While we had a strict protocol for salivary oxytocin collection, there was inherent risk to the samples arising from remote collection. Samples were stored in participants’ freezers for days to months prior to being collected and stored in our −80 °C laboratory freezer. While the typical home freezer is set at −18 °C, there may be variations across households as well as collection-to-storage times, leading to oxytocin degradation. We did, however, retain the vast majority of the samples (only 4/67 were unsuitable for analysis) and found expected differences in oxytocin levels according to breastfeeding status, supporting the validity of the data. 

### 4.5. Conclusions

Overall, our study found that new mothers with depression report more parenting-related stress and impairments in bonding compared to mothers without depression. These differences were not reflected in observations of mother–infant interactions or salivary oxytocin levels, suggesting that many of the building blocks of a strong mother–child relationship are there early in infancy regardless of depression status. Our results suggest a critical window for early intervention to support mothers as they form early bonds with their infants. It is imperative that postpartum depression is properly diagnosed and promptly treated, not only to help the new mother function at a critical stage of identity development, but to invest in the mother–infant dyad, leading to improved outcomes for baby. We will continue to follow these mothers, some of whom chose to begin SSRI treatment and others who did not, to determine the degree to which these patterns persist later in infancy and how they may vary based on changes in depression symptoms.

## Figures and Tables

**Table 1:** Demographic information by group.

Characteristic	Depressed (*n* = 45)	Controls (*n* = 25)	*p*-Value
Infant Sex			0.544
Female	20 (44%)	12 (48%)	
Male	25 (56%)	13 (52%)	
Marital Status			0.005
Married	30 (67%)	24 (96%)	
Cohabitating	6 (13%)	1 (4%)	
Divorced/separated	4 (9%)	0 (0%)	
Single	5 (11%)	0 (0%)	
Race			0.431
White	28 (62%)	18 (72%)	
Asian/Asian American	11 (24%)	5 (20%)	
Black/African American	6 (13%)	1 (4%)	
Multi-racial	0 (0%)	1 (4%)	
Ethnicity			1.00
Non-Hispanic	36 (80%)	20 (80%)	
Hispanic	9 (20%)	5 (20%)	
Maternal Education			0.056
High school	6 (13%)	0 (0%)	
Associate degree/some college	3 (7%)	2 (8%)	
Four-year college degree	15 (33%)	9 (36%)	
Graduate or professional degree	21 (47%)	14 (56%)	
Feeding status			0.025
Breastfed	19 (42%)	14 (56%)	
Mixed-fed	18 (40%)	11 (44%)	
Formula-fed	8 (18%)	0 (0%)	
Infant age (mos)	3.21 (1.40)	3.16 (1.28)	0.852
Depression symptoms (EPDS)	14.95 (3.95)	3.12 (1.28)	<0.001

*Note*. Chi-square tests were conducted to assess group differences in categorical variables. To maintain sufficient power to detect differences, groups were collapsed for analysis (marital status: married vs. not married; race: white vs. non-white; maternal education: college graduate vs. some college or less; feeding status: breastfeeding/mixed vs. formula). *T*-tests were used to assess differences in continuous variables.

**Table 2:** Descriptive information by group.

Measures *M (SD)*	Depressed (*n* = 45)	Controls (*n* = 25)	*p*-Value
Parenting stress (PSI-4-SF t-score)			
Parental Distress	63.27 (7.61)	43.96 (6.70)	<0.001
Parent–Child Dysfunctional Interaction	50.73 (9.14)	42.28 (5.00)	<0.001
Difficult Child	49.04 (10.98)	40.44 (5.83)	<0.001
Total Stress	55.44 (8.07)	41.56 (4.99)	<0.001
Bonding (PBQ raw score)			
Impaired Bonding	11.82 (6.89)	4.60 (3.27)	<0.001
Rejection and Anger	5.93 (4.47)	2.16 (2.46)	<0.001
Infant Focused Anxiety	6.29 (3.25)	2.56 (1.94)	<0.001
Free Play			
Maternal Sensitivity	4.57 (0.72)	4.70 (0.43)	0.814
Child Social Engagement	3.70 (0.68)	3.40 (1.00)	0.188
Dyadic Quality	3.92 (1.03)	3.94 (1.05)	0.929
Salivary oxytocin (pg/mL)	18.20 (14.51)	16.91 (15.25)	0.298

*Note*. One participant in the depressed group is missing free play data. Two participants in the depressed group and four in control group are missing oxytocin data. Group differences were assessed using *t*-tests. Due to violation of normality assumptions, differences in Maternal Sensitivity were assessed using Mann–Whitney U test. ANCOVA test, covarying for feeding status, was used for oxytocin comparison (estimated marginal means [standard error]: depressed = 19.06 [2.10]; controls = 15.20 [2.99]).

**Table 3:** COVID-19 questionnaire.

Measures *M (SD)*	Depressed (*n* = 45)	Controls (*n* = 25)	*p*-Value
*Because of COVID-19 (Likert Scale: 1: strongly disagree–5: strongly agree):*			
I have felt increased stress about food or supply shortages.	2.58 (1.59)	2.12 (1.30)	0.260
I have felt increased stress about returning to work after maternity leave due to exposure to the virus.	3.91 (1.38)	3.20 (1.63)	0.057
I have felt increased stress about childcare or taking care of children at home.	3.73 (1.53)	3.52 (1.50)	0.575
I have felt increased stress about losing a job or a decrease in family income	3.18 (1.64)	2.40 (1.56)	0.057

*Note*. *T*-tests were conducted to compare groups.

## Data Availability

The data presented in this study are available on request from the corresponding author (mcrichards@mednet.ucla.edu).
